# Supplementary figures and images for: In vivo two-photon microscopic observation and ablation in deeper brain regions realized by modifications of excitation beam diameter and immersion liquid
Source: PLoS One. 2020 Aug 7;15(8):e0237230. doi: 10.1371/journal.pone.0237230 (PMC7413496; doi:10.1371/journal.pone.0237230)

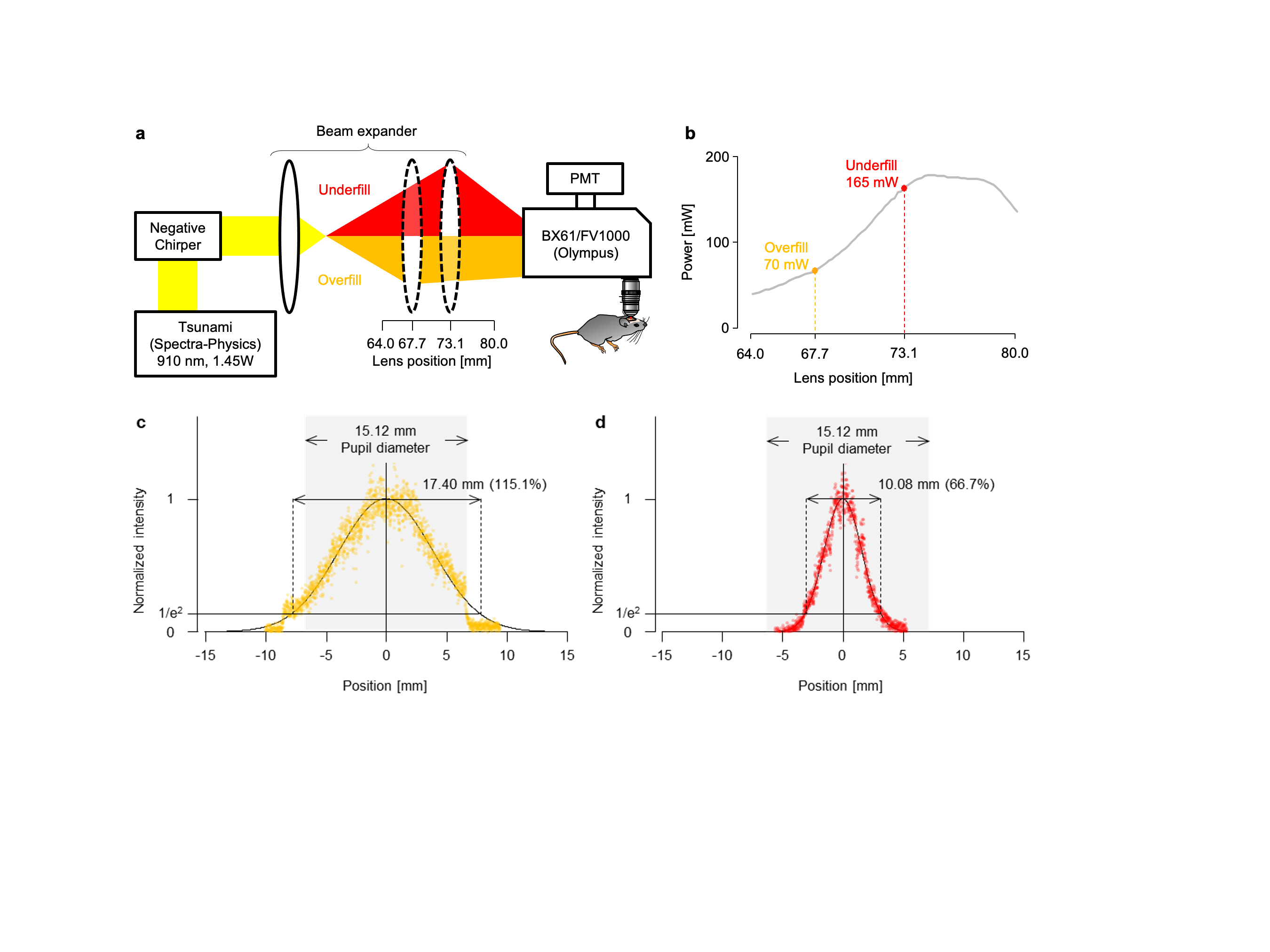

Supplement: S1 Fig — (a) Schematic illustration of the optical setup of the two-photon microscopy. (b) A graph showing the relationship between the position of the movable lens and the excitation laser power after the objective lens. (c, d) Measured intensity profiles of the excitation laser light beam before the pupil under (c) the overfilled condition and (d) the underfilled condition. (TIF) [file pone.0237230.s001.tif]

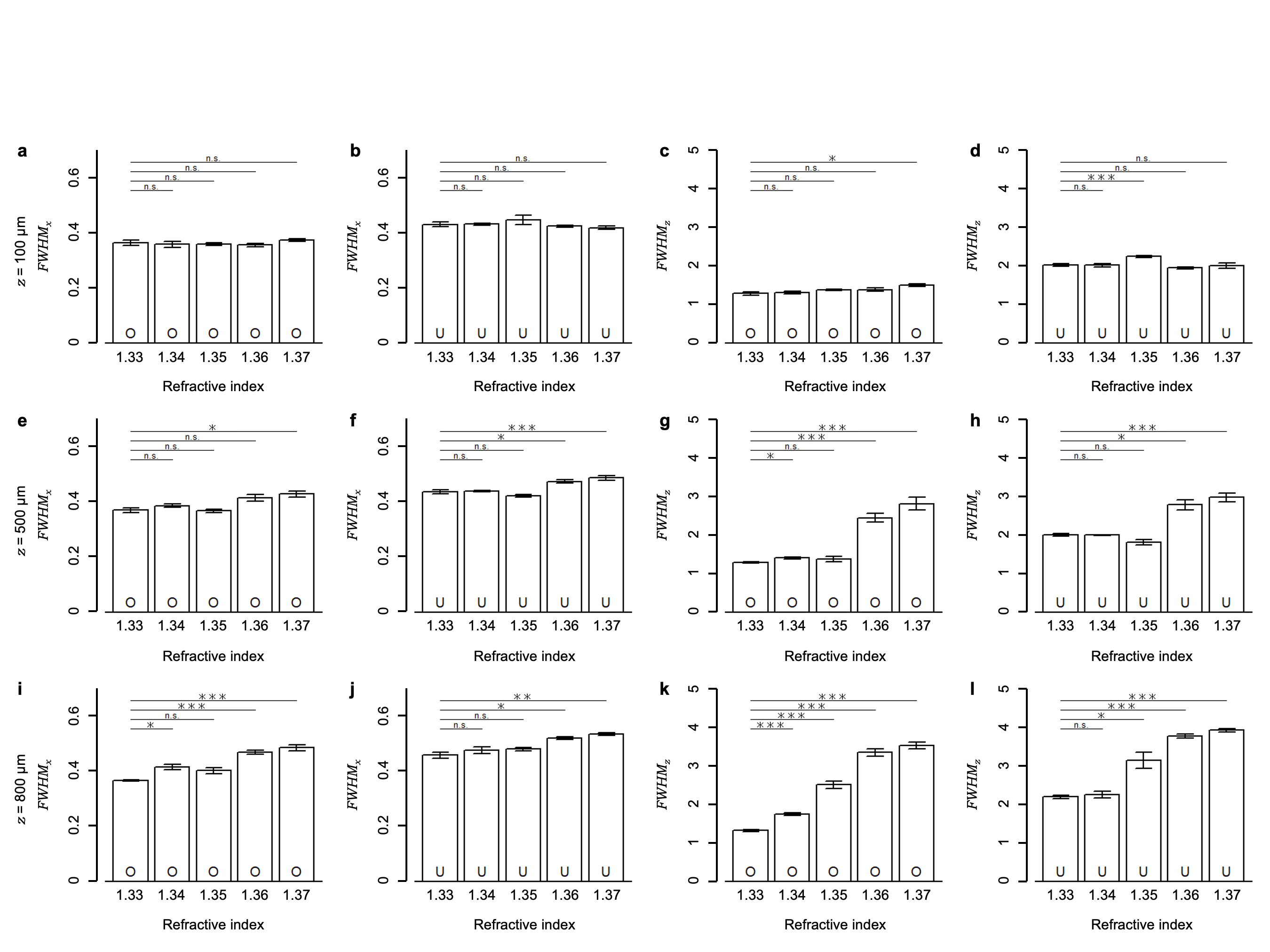

Supplement: S2 Fig — (a–h) Averaged FWHMs evaluated from the fluorescence intensity profiles of the beads embedded in the agarose-gels of which RIs were indicated as the white bar under each condition. All statistical tests were carried out at the RIs of the agarose gels with the other RI conditions. *: p < 0.05; **: p < 0.01; ***: p < 0.005 (Welch’s t-test with Bonferroni correction). Error bars represent s.e.m. (TIF) [file pone.0237230.s002.tif]

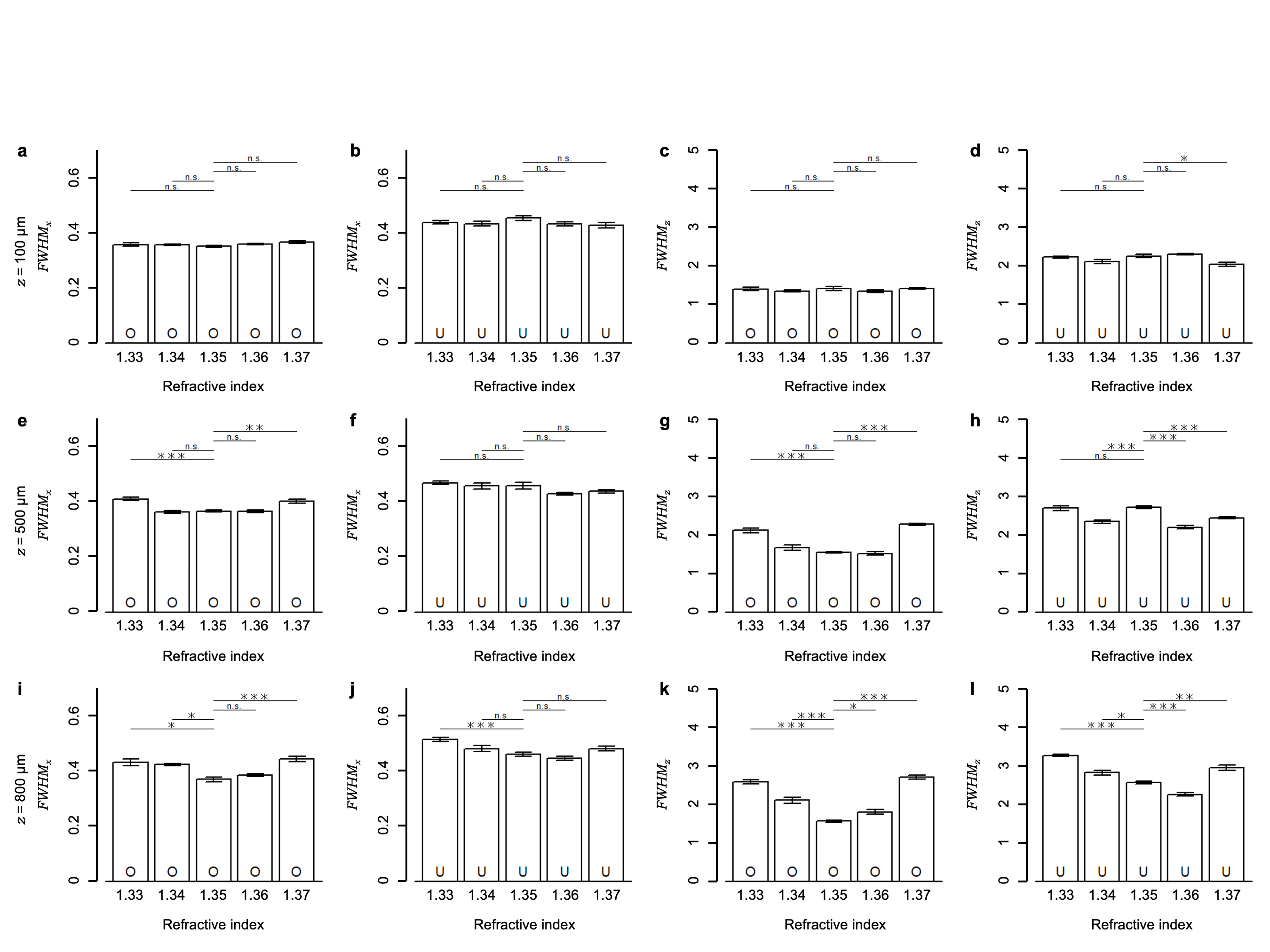

Supplement: S3 Fig — (a–h) Averaged FWHMs evaluated from the fluorescence intensity profiles of the beads embedded in the agarose-gels of which RIs were indicated as the white bar under each condition. All statistical tests were carried out at the RIs of agarose-gels with the other RI conditions. *: p < 0.05; **: p < 0.01; ***: p < 0.005 (Welch’s t-test with Bonferroni correction). Error bars represent s.e.m. (TIF) [file pone.0237230.s003.tif]

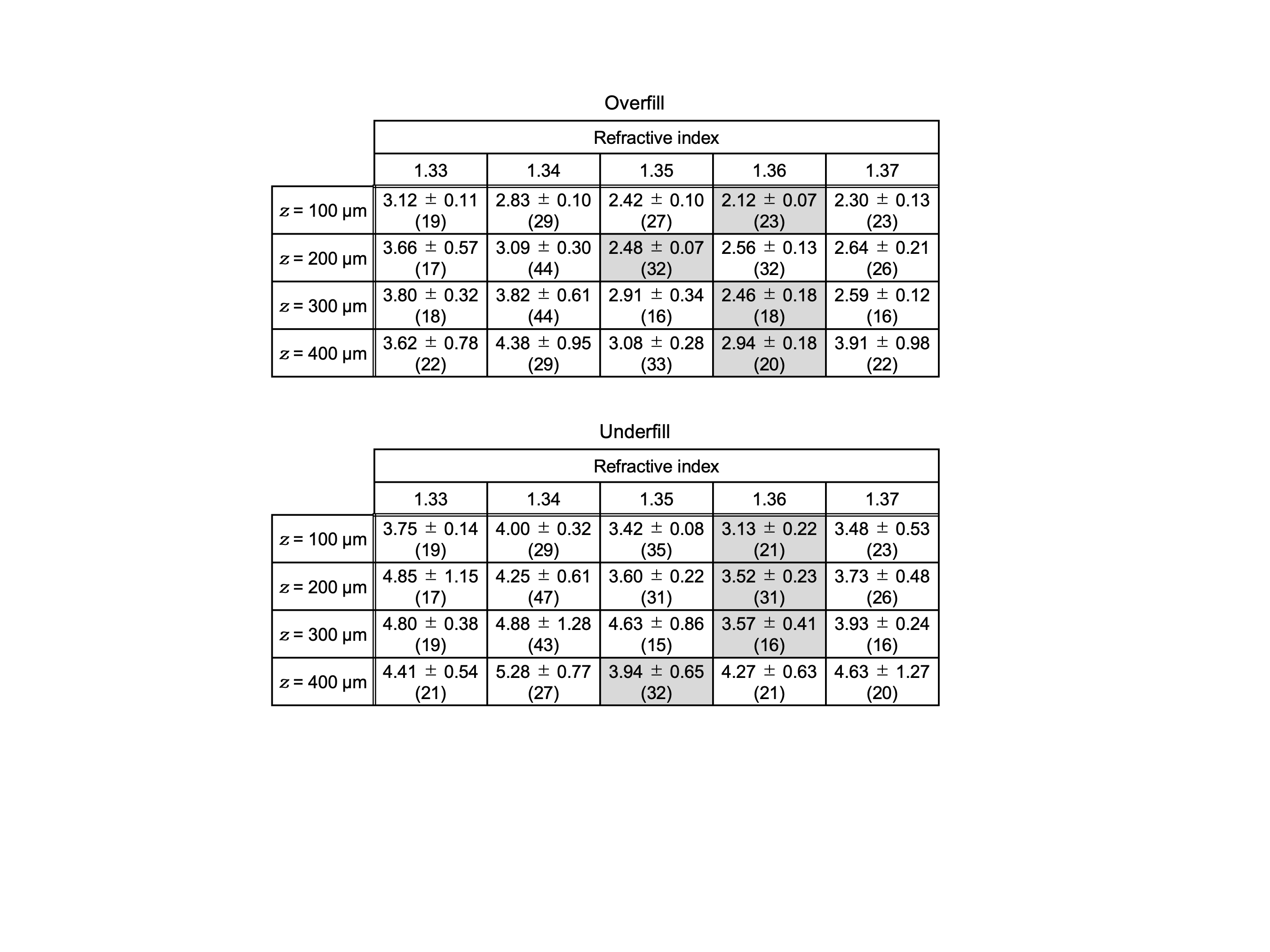

Supplement: S1 Table — FWHMz measured from intensity profiles of single microbeads obtained under each condition. All values represent mean ± s.e.m. Numbers in the parentheses represent the number of evaluated beads. Cells of gray background show the minimum FWHMs at each depth. (TIF) [file pone.0237230.s004.tif]

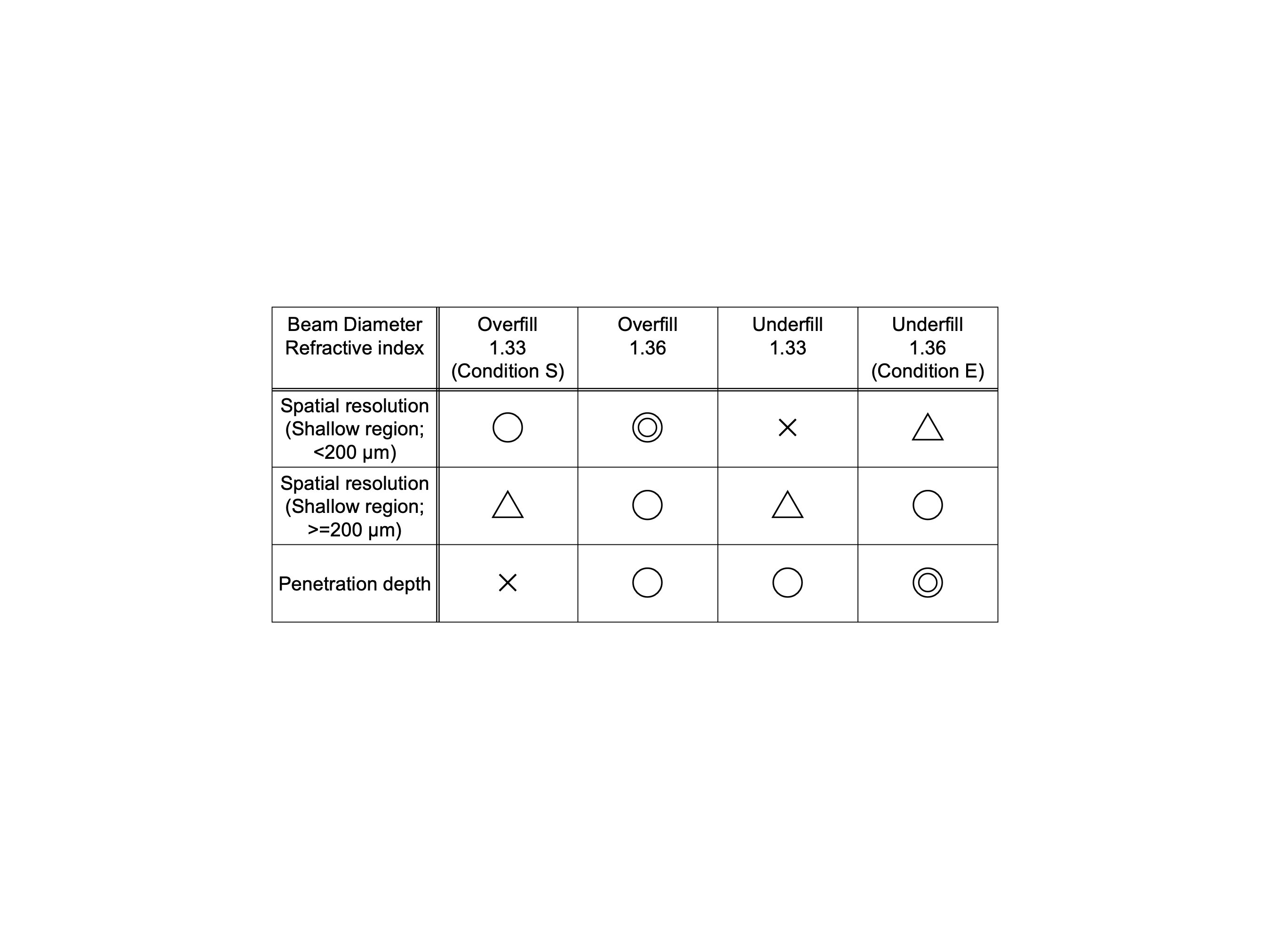

Supplement: S2 Table — Double circle, excellent; circle, good; triangle, average; cross, bad. (TIF) [file pone.0237230.s005.tif]
